# Clinical comparative analysis of endoscopic thyroidectomy via the thoracoareolar approach versus open surgery for the treatment of differentiated thyroid cancer

**DOI:** 10.1097/MD.0000000000046922

**Published:** 2026-01-23

**Authors:** Liangliang Huang, Shuang Li, Jianhong Shen, Jianping Yan, Lifu Su, Feng Song

**Affiliations:** a Nephrology Department, Hohhot First Hospital, Hohhot, Inner Mongolia, China; b Graduate School of Inner Mongolia Medical University, Hohhot, Inner Mongolia, China; c Cardiovascular Medicine Department, Hohhot First Hospital, Hohhot, Inner Mongolia, China; d Department of Thyroid, Breast and Hernia Surgery, Hohhot First Hospital, Hohhot, Inner Mongolia, China.

**Keywords:** complications, differentiated thyroid cancer, lumpectomy, open thyroidectomy, perioperative indicators, quality of life

## Abstract

The incidence of differentiated thyroid carcinoma has risen steadily in recent years, making it the most common thyroid malignancy. Surgical resection remains the primary treatment, but conventional open thyroidectomy (OT), while effective, often leaves prominent cervical scars that negatively affect aesthetics and quality-of-life. Endoscopic thyroidectomy (ET) via the thoracoareolar approach has been increasingly adopted, offering improved cosmetic outcomes and faster recovery, although evidence regarding its perioperative performance, complications, and follow-up outcomes remains limited. In this single-center retrospective cohort study, 150 patients with differentiated thyroid carcinoma treated between January 2020 and August 2023 were analyzed, including 70 who underwent ET and 80 who underwent OT. Baseline characteristics were comparable between groups. ET was associated with longer operative time but less intraoperative blood loss, shorter incisions, and more favorable postoperative recovery parameters, including earlier extubation, reduced hospital stay, and lower pain scores. Complication rates and the incidence of local recurrence or distant metastasis over 12 to 24 months did not differ significantly between groups, whereas quality-of-life scores (QLQ-C30) were significantly higher in the ET group. These findings suggest that thoracoareolar ET provides oncologic safety comparable to OT while conferring advantages in minimizing surgical trauma, accelerating recovery, and improving quality of life. ET may be particularly suitable for younger patients with greater cosmetic concerns, and further multicenter prospective studies are warranted to confirm its long-term efficacy and cost-effectiveness.

## 1. Introduction

In recent decades, the global incidence of thyroid cancer has shown a steady upward trend, with differentiated thyroid carcinoma (DTC) accounting for over 90% of cases and representing the predominant subtype of thyroid malignancy.^[1]^ Advances in diagnostic technology and the widespread use of ultrasound screening have markedly increased the detection of early microcarcinomas, contributing to the rising incidence. However, this growth has not been accompanied by a parallel increase in morbidity or mortality.^[2]^ Within current therapeutic strategies, surgery remains the cornerstone of DTC management.^[3]^ Conventional anterior cervical incision thyroidectomy (OT) allows for complete tumor resection but inevitably leaves visible scarring on the neck. For younger female patients in particular, such scars may not only compromise cosmetic outcomes but also lead to psychological distress and a reduced quality of life.^[4]^ Consequently, optimizing both aesthetic outcomes and quality of life while maintaining oncologic efficacy has become an important focus of contemporary clinical research.

With the advancement of minimally invasive techniques, endoscopic thyroidectomy (ET) has been increasingly adopted in clinical practice. Several approaches have been developed, including axillary, thoracic, areolar, and transoral vestibular access. Among these, the transareolar and thoracoareolar combined approaches have gained particular attention due to their concealed incisions and favorable cosmetic outcomes.^[5]^ Tan et al reported that, under strict patient selection, the transareolar approach achieved comparable surgical safety and oncologic clearance to conventional open thyroidectomy (OT).^[6]^ Similarly, a meta-analysis confirmed that ET was not inferior to OT regarding surgical margins, lymph node dissection, and other oncological parameters.^[7]^ Nevertheless, ET is generally associated with longer operative times and is subject to a considerable learning curve.^[8]^

Beyond surgical efficacy, increasing emphasis has been placed on postoperative recovery and quality of life (QoL). Kasemsiri et al compared transoral ET via the vestibular approach (TOETVA) with OT and found that patients undergoing ET reported significantly higher QoL scores at 2, 6, and 12 weeks postoperatively.^[9]^ Wu et al further demonstrated that ET yielded greater scar satisfaction and superior thyroid cancer–specific QoL scores, although no significant difference was observed in overall QoL.^[10]^ A systematic review also noted variability across studies regarding different QoL dimensions; however, most evidence indicated that ET offers advantages in cosmetic outcomes and postoperative pain control.^[11]^

Nonetheless, the current evidence comparing ET with OT remains insufficient. Most available studies are single-center, involve small sample sizes, and have limited follow-up, leaving uncertainties regarding long-term recurrence and distant metastasis.^[12]^ Moreover, the efficacy and safety of different ET approaches are heterogeneous. While some reviews have questioned the adequacy of central lymph node dissection and complication risks associated with the areolar or thoracoareolar approach,^[13]^ controlled trials have shown that, in experienced hands, the number of lymph nodes removed does not differ significantly between ET and OT.^[14]^ Furthermore, findings on postoperative quality-of-life are inconsistent, partly due to cultural variation and patient demographics. Therefore, high-quality, large-scale studies are needed to comprehensively evaluate the comparative efficacy, safety, and quality-of-life outcomes of ET and OT.

Building on these considerations, the present study aimed to compare thoracoareolar ET with conventional open surgery in patients with DTC. The comparison focused on perioperative parameters, postoperative recovery, complication rates, and follow-up quality-of-life. Through systematic data analysis and mid-term follow-up, we sought to further delineate the relative strengths and limitations of these 2 surgical approaches regarding oncologic control and quality-of-life outcomes, thereby providing evidence to inform clinical decision-making in surgical management.

## 2. Methodology

### 2.1. Study design and subjects

This study was approved by the Ethics Committee of Hohhot First Hospital. This single-center retrospective controlled study included 150 patients with pathologically confirmed DTC who underwent surgical treatment in our hospital between January 2020 and August 2023. Among them, 70 patients received laparoscopic thoracoareolar thyroidectomy, and 80 underwent conventional OT. All procedures were performed by experienced attending surgeons, each of whom had completed more than 100 endoscopic thyroidectomies, thereby ensuring surgical quality and the reliability of outcomes. The study protocol was approved by the hospital’s Ethics Committee, and written informed consent was obtained from all participants.

### 2.2. Inclusion and exclusion criteria

Inclusion criteria:

1. Postoperative pathological confirmation of DTC (papillary or follicular) according to WHO classification criteria.
2. Clinical staging of stage I–IV based on the 8th edition AJCC TNM system.
3. Age between 18 and 70 years.
4. Availability of complete clinical and follow-up data.

Exclusion criteria:

1. Coexistence of other thyroid malignancies or severe cervical deformities.
2. Presence of distant metastasis or requirement for combined resection of other organs.
3. History of prior neck surgery or radiotherapy.
4. Severe cardiopulmonary dysfunction or inability to tolerate anesthesia.
5. Severe psychiatric disorders or poor treatment compliance.
6. Incomplete follow-up data.

### 2.3. Surgical methods

#### 2.3.1. Endoscopic group

Under general anesthesia, ET via the thoracoareolar approach was performed. Surgical access was established through bilateral areolar margins and the anterior chest wall. Carbon dioxide insufflation was applied to maintain a clear operative field, with pressure controlled at 6 to 8 mm Hg. Thyroidectomy and central lymph node dissection were then carried out according to standard endoscopic procedures.

#### 2.3.2. Open group

A transverse anterior cervical incision was made to expose the thyroid gland and adjacent tissues, followed by tumor resection and central lymph node dissection.

In both groups, standard oncologic principles were strictly followed, with meticulous preservation of the recurrent laryngeal nerve and parathyroid gland function.

### 2.4. Observation indicators

**General information:** age, sex, tumor diameter, pathological type, and TNM stage.

**Surgical parameters:** operative time, intraoperative blood loss, incision length, and number of lymph nodes dissected.

**Postoperative recovery:** 24-h drainage volume, time to extubation, length of hospital stay, and 24-h pain score (visual analogue scale [VAS]), 0–10).

**Complications:** recurrent laryngeal nerve injury, hypocalcemia, postoperative hemorrhage, and wound infection; overall complication rate was calculated.

**Follow-up outcomes:** local recurrence and distant metastasis during 12 to 24 months of follow-up; quality-of-life was evaluated using the EORTC QLQ-C30 (version 3) questionnaire, with standardized scores ranging from 0 to 100, higher scores reflecting better quality-of-life.

### 2.5. Follow-up method

Follow-up was conducted through a combination of outpatient visits and telephone contacts, with a completion rate of 100%. Assessments included neck ultrasonography and thyroid function tests, with additional CT imaging performed if recurrence or metastasis was clinically or radiologically suspected. Quality of life was evaluated using self-administered questionnaires completed by patients during follow-up visits.

### 2.6. Statistical analysis

All statistical analyses were performed using SPSS version 26.0. Normality of continuous variables was assessed with the Shapiro–Wilk test, and homogeneity of variance with Levene test. Data following a normal distribution with equal variance were presented as mean ± standard deviation (x̄ ± s) and compared using the independent-samples *t*-test; otherwise, the Mann–Whitney *U* test was applied. Categorical variables were expressed as counts and percentages, with group comparisons performed using the χ² test. Because this was a retrospective study, no a priori sample size calculation was performed. A post hoc power analysis showed that, with α = 0.05 and sample sizes of 70 and 80, the study had > 0.99 power to detect observed differences in operative time, blood loss, and postoperative pain, but limited power for the low-incidence complications. All tests were 2-sided, and a *P*-value < .05 was considered statistically significant.

## 3. Results

### 3.1. Comparison of general information

A total of 150 patients with DTC were enrolled, with 70 in the endoscopic group and 80 in the open group. Baseline characteristics, including age, sex, tumor diameter, pathological type, and TNM stage, showed no significant differences between groups (all *P* > .05), indicating good comparability (Table 1).

**Table 1:** Comparison of baseline characteristics between patients undergoing thoracoareolar ET and OT.

Projects	Endoscopic group (N = 70)	Open group (N = 80)	χ²/t	*P*
Age (years, x̄±s)	39.5 ± 9.8	41.2 ± 10.1	−1.045	.298
Sex (male/female)	18/52	22/58	0.004	.951
Tumor diameter (cm, x̄±s)	1.5 ± 0.6	1.6 ± 0.7	−0.942	.348
Pathologic type (papillary/follicular)	65/5	73/7	0.004	.952
TNM staging (I–II/III–IV)	62/8	69/11	0.033	.857

ET = endoscopic thyroidectomy, OT = open thyroidectomy.

### 3.2. Surgery-related indexes

The laparoscopic group had a significantly longer operative time compared with the open group (140.5 ± 25.3 minutes vs 115.7 ± 20.6 minutes, *P* < .001). However, intraoperative blood loss was lower (50.8 ± 18.2 mL vs 70.5 ± 22.6 mL, *P* < .001), and the incision length was markedly shorter (3.2 ± 0.8 cm vs 7.8 ± 1.2 cm, *P* < .001). No significant difference was observed in the number of dissected lymph nodes between the 2 groups (*P* = .405) (Table 2). These findings indicate that lumpectomy may offer advantages in minimizing surgical trauma.

**Table 2:** Comparison of surgical parameters between lumpectomy and OT in patients with DTC.

Projects	Endoscopic group (N = 70)	Open group (N = 80)	*t*	*P*
Operative time (min, x̄±s)	140.5 ± 25.3	115.7 ± 20.6	6.524	<.001
Intraoperative bleeding (mL, x̄±s)	50.8 ± 18.2	70.5 ± 22.6	−5.909	<.001
Length of incision (cm, x̄±s)	3.2 ± 0.8	7.8 ± 1.2	−27.921	<.001
Number of lymph nodes cleared (pcs, x̄±s)	6.5 ± 2.1	6.8 ± 2.3	−0.835	.405

DTC = differentiated thyroid carcinoma, OT = open thyroidectomy.

### 3.3. Postoperative recovery

Postoperative recovery outcomes were more favorable in the endoscopic group compared with the open group. Patients undergoing lumpectomy had reduced drainage volume (85.2 ± 25.7 mL vs 95.6 ± 28.3 mL, *P* = .020), earlier extubation (2.5 ± 0.7 days vs 3.0 ± 0.8 days, *P* < .001), shorter hospital stay (5.6 ± 1.5 days vs 6.8 ± 1.8 days, *P* < .001), and lower postoperative pain scores (VAS: 2.8 ± 0.9 vs 4.2 ± 1.0, *P* < .001) (Table 3). These findings indicate that lumpectomy may accelerate recovery and enhance patient comfort.

**Table 3:** Comparison of postoperative recovery between laparoscopic and OT.

Projects	Endoscopic group (N = 70)	Open Group (N = 80)	*t*	*P*
Drainage volume (mL, x̄±s)	85.2 ± 25.7	95.6 ± 28.3	−2.358	.020
Time to extubation (d, x̄±s)	2.5 ± 0.7	3.0 ± 0.8	−4.082	<.001
Length of hospitalization (d, x̄±s)	5.6 ± 1.5	6.8 ± 1.8	−4.452	<.001
Postoperative pain score (VAS, 0–10)	2.8 ± 0.9	4.2 ± 1.0	−9.024	<.001

OT = open thyroidectomy, VAS = visual analogue scale.

### 3.4. Complication rates

No significant differences were observed between the 2 groups in the incidence of recurrent laryngeal nerve injury, hypocalcemia, postoperative bleeding, or wound infection (all *P* > .05). The overall complication rate was 12.9% in the endoscopic group and 18.8% in the open group, with no statistical significance (*P* = .448) (Table 4). These results suggest that both procedures offer comparable safety profiles.

**Table 4:** Comparison of postoperative complications between lumpectomy and open groups.

Projects	Endoscopic group (N = 70)	Open group (N = 80)	χ²	*P*
Laryngeal recurrent nerve injury	2 (2.9%)	3 (3.8%)	0.001	.999
Hypocalcemia	5 (7.1%)	7 (8.8%)	0.004	.952
Postoperative bleeding	1 (1.4%)	2 (2.5%)	0.001	.999
Incision infection	1 (1.4%)	3 (3.8%)	0.139	.71
Overall complication rate	9 (12.9%)	15 (18.8%)	0.576	.448

### 3.5. Follow-up results

During the 12 to 24 month follow-up, no significant differences were observed between the 2 groups in local recurrence (1.4% vs 2.5%) or distant metastasis (0 vs 1.3%) (both *P* = .999), indicating comparable tumor control. However, quality-of-life scores (QLQ-C30) were significantly higher in the endoscopic group than in the open group (82.5 ± 8.3 vs 78.2 ± 9.1, *P* = .003) (Table 5), suggesting that lumpectomy provides a long-term advantage in quality-of-life improvement.

**Table 5:** Comparison of 12 to 24 month follow-up outcomes between lumpectomy and OT.

Projects	Endoscopic group (N = 70)	Open group (N = 80)	χ²	*P*
Local recurrence	1 (1.4%)	2 (2.5%)	0.001	.999
Distant metastasis	0 (0%)	1 (1.3%)	0.001	.999
Quality of life score (QLQ-C30, total score, x̄±s)	82.5 ± 8.3	78.2 ± 9.1	3.026	.003

OT = open thyroidectomy

## 4. Discussion

In this study, we compared the efficacy and safety of ET via the thoracoareolar approach with conventional OT in patients with DTC. ET was associated with a longer operative time but demonstrated advantages over OT in terms of reduced intraoperative blood loss, shorter incision length, lower postoperative drainage, earlier extubation, shorter hospital stay, and better pain control. Complication rates did not differ significantly between groups, and both procedures achieved comparable local recurrence and distant metastasis rates during the 12 to 24 month follow-up. However, quality-of-life scores were significantly higher in the ET group. Overall, these findings indicate that ET provides the combined benefits of reduced surgical trauma, faster recovery, and improved quality-of-life without compromising oncologic safety.

Several studies have reported that ET requires longer operative time than OT, primarily due to the technical complexity of creating the working space, instrument access, and the surgical team’s level of expertise.^[15,16]^ The findings of our study are consistent with these observations. However, operative time tends to decrease with accumulated experience and standardization of techniques. Moreover, the influence of the learning curve highlights the importance of surgeon training and effective teamwork in the implementation of ET to mitigate the adverse effects of limited experience.

With respect to intraoperative trauma, our study showed that mean blood loss in the ET group was 50.8 mL, significantly lower than the 70.5 mL observed in the OT group. Similar findings have been reported in other controlled studies. The magnified visualization and precise coagulation provided by energy devices in ET help minimize vascular injury, thereby reducing intraoperative bleeding.^[17,18]^ In addition, the incision length is reduced to about 3 cm, with scars concealed within the areolar or chest region, offering clear cosmetic advantages over OT. This benefit is particularly relevant given the potential long-term psychosocial impact of visible neck scarring.

Regarding postoperative recovery, our study showed that the ET group had shorter extubation time (2.5 days), reduced hospital stay (5.6 days), and lower pain scores (VAS: 2.8) compared with the OT group. These results align with findings from previous prospective studies and systematic reviews, which demonstrated that minimally invasive thyroidectomy reduces analgesic requirements and facilitates faster recovery.^[19–21]^ Together with the growing adoption of enhanced recovery after surgery protocols, the recovery benefits of ET are even more pronounced, underscoring its suitability within modern surgical management.

With respect to complications, our study found rates of 2.9% for recurrent laryngeal nerve injury, 7.1% for hypocalcemia, and an overall complication rate of 12.9% in the ET group, which were comparable to those in the OT group and within the 2% to 18% range reported in the literature.^[22]^ Evidence from large databases and multicenter studies has similarly confirmed that ET and OT have comparable complication rates, without an increased risk of recurrent laryngeal nerve or parathyroid injury.^[14,23,24]^ Although some investigators have raised concerns regarding the adequacy of central lymph node dissection in ET, multiple studies have demonstrated that, in experienced teams, the number of nodes removed and oncologic outcomes do not differ significantly between ET and OT.^[14,25]^ This is consistent with our findings, as no significant difference in the number of dissected lymph nodes was observed between the 2 groups.

Regarding tumor control, the ET group in our study showed a local recurrence rate of 1.4% and no distant metastasis during 12 to 24 months of follow-up, comparable to outcomes in the OT group, indicating a favorable mid-term oncologic safety profile. Consistently, a recent multicenter study reported that ET was not inferior to OT in terms of recurrence and 5-year survival.^[25]^ A systematic review further concluded that ET is comparable to OT in patients with low- to intermediate-risk DTC, but should be applied cautiously in high-risk cases, such as those with large tumors or invasion of adjacent structures.^[26]^ Taken together, these findings suggest that ET is a safe and feasible surgical option when applied under strict indications.

Quality of life is a key endpoint in evaluating the value of modern surgical techniques. In our study, the quality-of-life score in the ET group (82.5) was significantly higher than that in the OT group (78.2), and this difference persisted at 12 to 24 months of follow-up, indicating sustained benefit. Previous prospective studies and systematic reviews have likewise shown that minimally invasive approaches provide advantages over conventional surgery in scar aesthetics, pain relief, and social functioning.^[19,22,26]^ These findings are particularly relevant for young female patients, for whom cosmetic outcomes and psychological well-being are major concerns, suggesting that ET offers not only short-term benefits but also potential long-term improvements in quality-of-life.

This study has several limitations. First, as a single-center retrospective analysis, it is inherently subject to selection bias and information bias, which may have influenced patient inclusion, data completeness, and the accuracy of outcome assessment. Although efforts were made to ensure comparable baseline characteristics and standardized surgical techniques, these biases could still affect the generalizability and internal validity of the results. Second, the relatively short follow-up period limited the evaluation of long-term recurrence and survival outcomes. Third, cosmetic satisfaction questionnaires and cost-effectiveness assessments were not included, restricting a comprehensive evaluation of the overall value of ET. Future studies should therefore adopt a prospective, multicenter randomized design with larger sample sizes and extended follow-up durations. Such studies should also incorporate standardized quality-of-life, cosmetic, and economic evaluation tools to further validate and strengthen the present findings.

In conclusion, this study shows that lumpectomy thyroidectomy via the thoracoareolar approach offers reduced surgical trauma, faster recovery, and improved quality-of-life, while maintaining oncologic safety in patients with DTC. The procedure is especially beneficial for younger patients who place greater emphasis on cosmetic outcomes. With continued technological refinement and growing surgical expertise, ET is likely to see broader clinical adoption; however, high-quality studies are still needed to confirm its long-term efficacy and cost-effectiveness.

## Author contributions

**Conceptualization:** Liangliang Huang, Shuang Li, Jianhong Shen, Lifu Su, Feng Song.

**Data curation:** Liangliang Huang, Shuang Li, Jianping Yan, Lifu Su, Feng Song.

**Formal analysis:** Liangliang Huang, Jianhong Shen, Jianping Yan, Feng Song.

**Funding acquisition:** Shuang Li, Feng Song.

**Investigation:** Feng Song.

**Writing – original draft:** Liangliang Huang, Lifu Su, Feng Song.

**Writing – review & editing:** Liangliang Huang, Jianping Yan, Lifu Su, Feng Song.
